# ECG challenge: a slower-than-usual heart transplant

**DOI:** 10.1093/ehjcr/ytaf635

**Published:** 2025-12-06

**Authors:** Miguel Sáenz-Molina, Alejandro Carta-Bergaz, Javier Castrodeza

**Affiliations:** Instituto de Investigación Sanitaria Gregorio Marañón (IiSGM), Calle Doctor Esquerdo 46, Madrid 28007, Spain; Servicio de Cardiología, Hospital General Universitario Gregorio Marañón, Calle Doctor Esquerdo 46, Madrid 28007, Spain; Centro de Investigación Biomédica en Red (CIBERCV), Madrid, Spain; Instituto de Investigación Sanitaria Gregorio Marañón (IiSGM), Calle Doctor Esquerdo 46, Madrid 28007, Spain; Servicio de Cardiología, Hospital General Universitario Gregorio Marañón, Calle Doctor Esquerdo 46, Madrid 28007, Spain; Centro de Investigación Biomédica en Red (CIBERCV), Madrid, Spain; Facultad de Medicina, Universidad Complutense, Plaza Ramón y Cajal s/n, 28040 Madrid, Spain; Instituto de Investigación Sanitaria Gregorio Marañón (IiSGM), Calle Doctor Esquerdo 46, Madrid 28007, Spain; Servicio de Cardiología, Hospital General Universitario Gregorio Marañón, Calle Doctor Esquerdo 46, Madrid 28007, Spain; Centro de Investigación Biomédica en Red (CIBERCV), Madrid, Spain; Facultad de Medicina, Universidad Complutense, Plaza Ramón y Cajal s/n, 28040 Madrid, Spain

**Keywords:** Heart transplantation, Sinus node dysfunction, Pacemaker

## Case presentation

**Figure 1:**
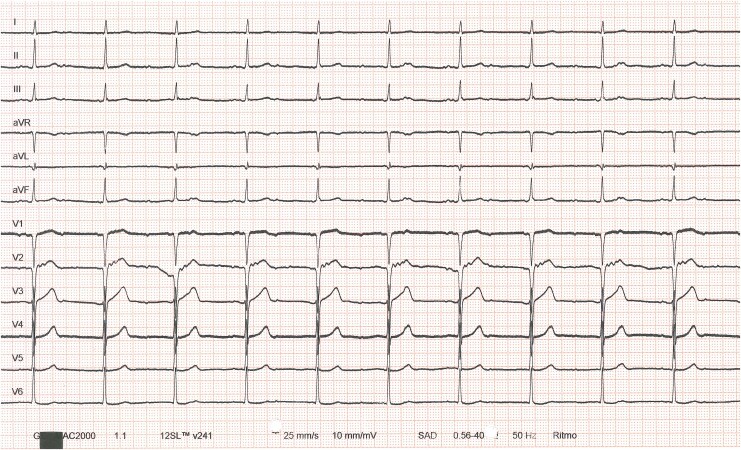
Twelve-lead ECG of the patient following orthotopic heart transplantation.

A 50-year-old male underwent orthotopic heart transplantation for arrhythmogenic cardiomyopathy with biventricular involvement and refractory ventricular arrhythmias. The graft was obtained from a donation after circulatory death from a male donor with no known cardiac disease and a normal pre-procurement ECG. After transplantation, the patient exhibited minimal heart rate variability, with nocturnal rates as low as 30 bpm and daytime rates not exceeding 45 bpm. He reported dyspnoea at rest without clinical signs of heart failure. Haemodynamic assessment during the second postoperative week showed normal filling pressures and cardiac output, with no evidence of rejection. Because of suspected chronotropic incompetence, an isoproterenol infusion was initiated, increasing the resting heart rate to 70 bpm and improving symptoms. After β-agonist withdrawal in the fifth postoperative week, left-sided filling pressures rose. Endomyocardial biopsy was normal without acute rejection. A 12-lead ECG was obtained and is presented in *Figure 1*.

## Question 1- Which of the following statements regarding the ECG is correct?

- Transient post-transplant sinus node dysfunction without need for intervention.
- Complete atrioventricular block with ventricular escape rhythm.
- Atrial dissociation due to dual *P* waves in a bi-atrial (Shumway-type) heart transplantation.
- Accelerated junctional rhythm following bicaval heart transplantation.
- Multifocal atrial rhythms following bicaval heart transplantation.

**Correct answer: Option (c)**

## Discussion and explanation

This ECG belongs to a patient who underwent orthotopic heart transplantation using the Shumway bi-atrial technique. The native atrial cuff—including the recipient sinus node—is electrically isolated from the donor atrium by the surgical suture line. Broad, notched *P* waves from the diseased recipient atrium (see Supplementary material online, *Figure S1*, red circles) are dissociated from the QRS complexes, which arise from a narrow-QRS junctional escape rhythm in the donor heart. In lead V2, a small retrograde notch (see Supplementary material online, *Figure S1*, green arrows), intermittently observed on the rhythm strip and absent during donor atrial pacing (see Supplementary material online, *Figure S2*), supports this interpretation. Therefore, the tracing demonstrates atrial dissociation, not complete atrioventricular block.

## Question 2 – Which of the following is the most relevant risk factor of sinus node disfunction in this patient?

- Orthotopic heart transplantation using the Shumway bi-atrial technique.
- Recipient age ≥ 40 years.
- The use of beta blockers before transplantation.
- Male donor.
- Arrhythmogenic cardiomyopathy as the cause for transplantation.

**Correct answer: Option (c)**

## Discussion and explanation

Compared with the Shumway technique, the bicaval surgical technique is associated with a substantially lower need for postoperative pacemaker requirement.^1^

In post-transplant patients with the Shumway technique who were also previously treated with class III antiarrhythmic drugs (such as amiodarone or sotalol),^2^ significant sinus node dysfunction is not uncommon and may allow the emergence of ectopic automatic foci, as in the present case, where a nodal escape rhythm was identified.

## Question 3 – What is the next appropriate step in management?

- Wait and see, sinus node disfunction is commonly reversible in cardiac transplant after 5 weeks.
- Implantation of an AAI pacemaker.
- Wait and repeat the haemodynamic assessment by right heart catheterization before the implantation of a cardiac device.
- Implantation of a VVI pacemaker.
- Assessment of the HV interval to decide whether a pacemaker is necessary.

**Correct answer: option (b)**

## Discussion and explanation

Because the patient remained dependent on chronotropic support 5 weeks after transplantation with high filling left-sided pressures, atrial pacing was indicated.^3^

As atrioventricular conduction was preserved, a single chamber pacemaker was implanted. After the implantation of an AAI pacemaker, the follow-up ECG showed a narrow-paced *P* wave (see Supplementary material online, *Figure S2*, green circles) with 1:1 atrioventricular conduction in the donor heart, while the recipient’s broad, notched *P* waves (see Supplementary material online, *Figure S2*, red circle) persisted and remained dissociated.

## Supplementary Material

ytaf635_Supplementary_Data

## Data Availability

All data underlying this article are included within the article and its Supplementary material.

## Notes

**Consent:** Written informed consent for publication was obtained from the patient in accordance with COPE and EHJ–Case Reports guidelines.

**Funding:** None declared.
