# Supplementary material for: ECG challenge: a slower-than-usual heart transplant
Source: Eur Heart J Case Rep. 2025 Dec 6;9(12):ytaf635. doi: 10.1093/ehjcr/ytaf635 (PMC12715497; doi:10.1093/ehjcr/ytaf635)

**SUPPLEMENTARY MATERIAL**

**Supplementary material, Figure S1**. **Atrial dissociation after Shumway bi-atrial heart transplantation**. Broad, notched P waves from the recipient atrial remnant (red circles) are dissociated from the narrow QRS complexes. A small notch in lead V2 (green arrows) reflects retrograde activation of the donor atrium from a junctional focus. The schematic (upper right) illustrates dual atrial activity—recipient sinus node (SAN-r, red) isolated from donor atrium and donor sinus node (SAN-d) suppressed by junctional rhythm (AVN, green).


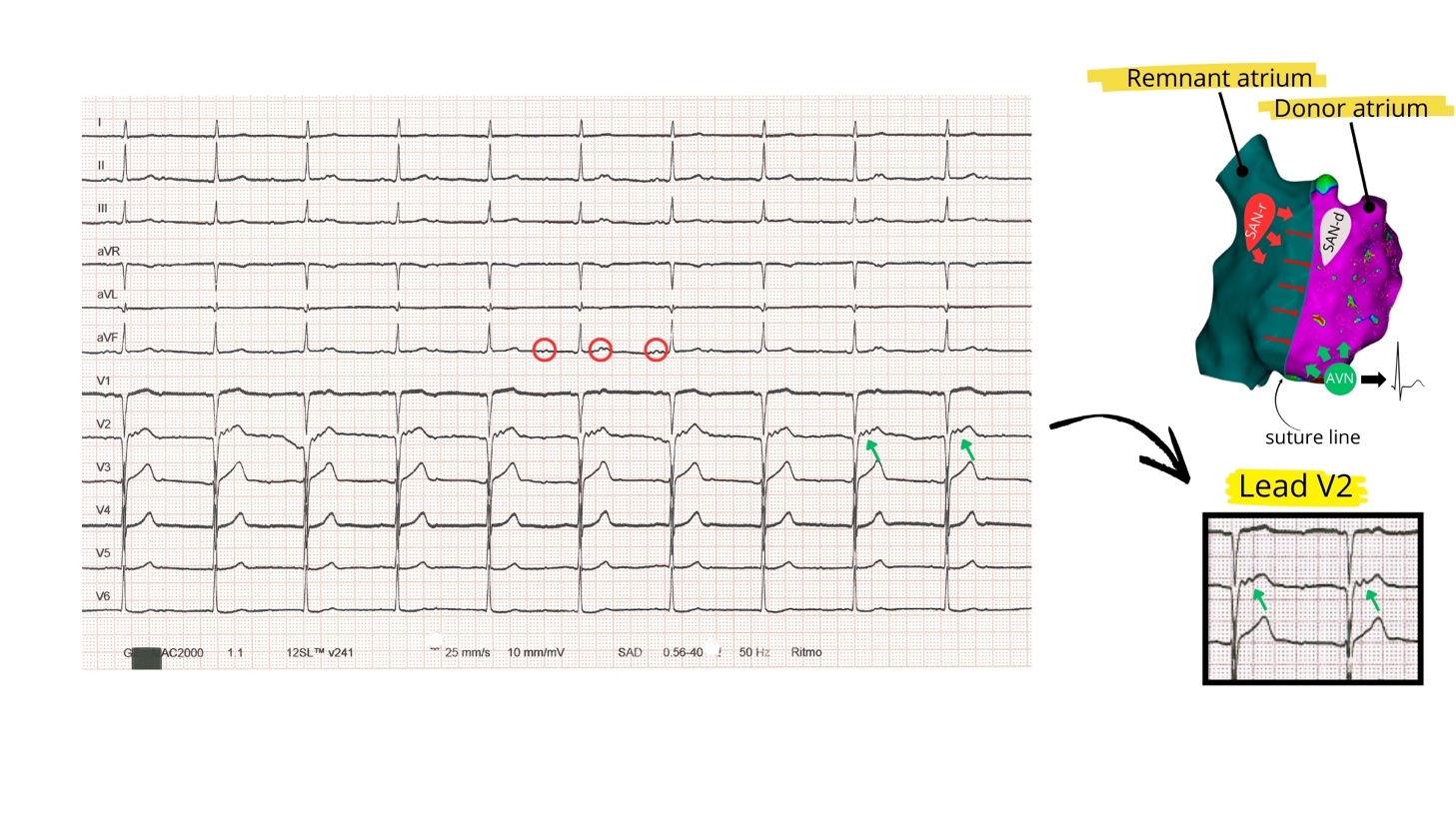


**Supplementary material, Figure S2. AAI pacing of the donor atrium restores 1:1 atrioventricular conduction**. Narrow paced P waves (green circles) originate from the donor atrium, while broad, dissociated P waves from the recipient atrial cuff (red circles) persist.


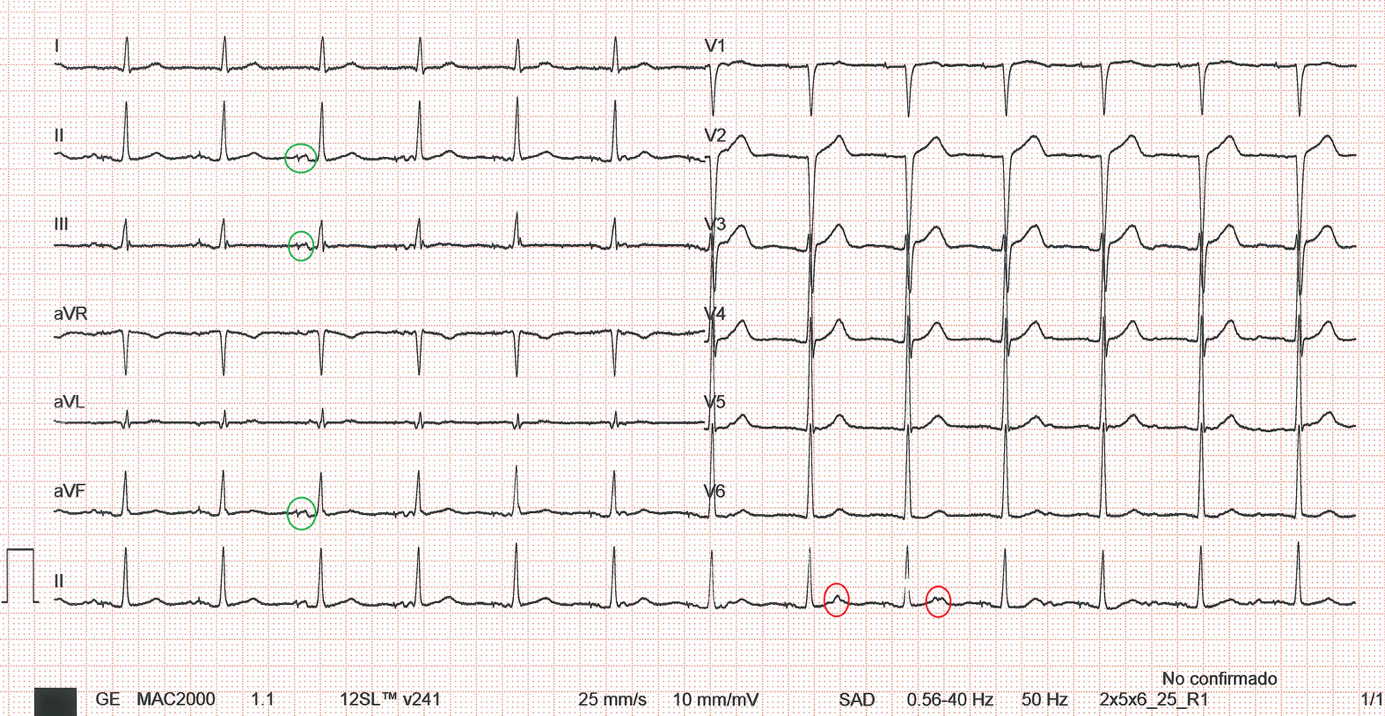

Supplement: ytaf635_Supplementary_Data [file ytaf635_supplementary_data.docx]
